# Dioxin Exposure and Cancer Risk in the Seveso Women’s Health Study

**DOI:** 10.1289/ehp.1103720

**Published:** 2011-08-02

**Authors:** Marcella Warner, Paolo Mocarelli, Steven Samuels, Larry Needham, Paolo Brambilla, Brenda Eskenazi

**Affiliations:** 1 Center for Environmental Research and Children’s Health, School of Public Health, University of California at Berkeley, Berkeley, California, USA; 2 Department of Laboratory Medicine, University of Milano-Bicocca, School of Medicine, Hospital of Desio, Desio-Milano, Italy; 3 State University of New York, Albany, New York, USA; 4 Division of Environmental Health Laboratory Science, National Center for Environmental Health, Centers for Disease Control and Prevention, Atlanta, Georgia, USA

**Keywords:** cancer, cohort studies, dioxins, environmental carcinogens, female, neoplasms, tetrachlorodibenzodioxin

## Abstract

Background: 2,3,7,8-Tetrachlorodibenzo-*para*-dioxin (TCDD), a widespread environmental contaminant, disrupts multiple endocrine pathways. The International Agency for Research on Cancer classified TCDD as a known human carcinogen, based on predominantly male occupational studies of increased mortality from all cancers combined.

Objectives: After a chemical explosion on 10 July 1976 in Seveso, Italy, residents experienced some of the highest levels of TCDD exposure in a human population. In 1996, we initiated the Seveso Women’s Health Study (SWHS), a retrospective cohort study of the reproductive health of the women. We previously reported a significant increased risk for breast cancer and a nonsignificant increased risk for all cancers combined with individual serum TCDD, but the cohort averaged only 40 years of age in 1996. Herein we report results for risk of cancer from a subsequent follow-up of the cohort in 2008.

Methods: In 1996, we enrolled 981 women who were 0–40 years of age in 1976, lived in the most contaminated areas, and had archived sera collected near the explosion. Individual TCDD concentration was measured in archived serum by high-resolution mass spectrometry. A total of 833 women participated in the 2008 follow-up study. We examined the relation of serum TCDD with cancer incidence using Cox proportional hazards models.

Results: In total, 66 (6.7%) women had been diagnosed with cancer. The adjusted hazard ratio (HR) associated with a 10-fold increase in serum TCDD for all cancers combined was significantly increased [adjusted HR = 1.80; 95% confidence interval (CI): 1.29, 2.52]. For breast cancer, the HR was increased, but not significantly (adjusted HR = 1.44; 95% CI: 0.89, 2.33).

Conclusions: Individual serum TCDD is significantly positively related with all cancer incidence in the SWHS cohort, more than 30 years later. This all-female study adds to the epidemiologic evidence that TCDD is a multisite carcinogen.

The compound, 2,3,7,8-tetrachlorodibenzo-*p*-dioxin (TCDD, or dioxin) is a widespread environmental contaminant (Birnbaum 1994, 1995; Zook and Rappe 1994). In animals, TCDD is a potent multisite carcinogen and has been shown to disrupt multiple endocrine pathways [Birnbaum 1994, 1995; Birnbaum and Fenton 2003; International Agency for Research on Cancer (IARC) 1997]. In 1997, the IARC classified TCDD as carcinogenic to humans (Group 1), based on limited evidence of carcinogenicity in humans, sufficient evidence in animals, and strong evidence in humans and animals for a common mechanism of action via initial binding to the aryl hydrocarbon receptor (AhR) (IARC 1997). Binding of the AhR leads to changes in gene expression, cell replication, and apoptosis. In 2009, IARC reconfirmed the classification of TCDD as a Group 1 carcinogen, citing sufficient epidemiological evidence for all cancers combined (Baan et al. 2009). This conclusion was based on male occupational cohort studies of increased mortality from all cancers combined, but no particular cancer sites were predominant.

Animal studies report a higher incidence of tumors associated with TCDD exposure in females than in males (Kociba et al. 1978). However, few studies in humans have investigated cancer in women associated with TCDD exposure. Two occupational cohort studies of workers employed in the production of chlorophenoxy herbicides have examined the relationship between TCDD exposure and cancer risk in females (Flesch-Janys et al. 1999; Kogevinas et al. 1993, 1997; Manz et al. 1991). Although limited by small sample size and lack of individual exposure data, both studies report increased risks for mortality or incidence from all cancers combined in the subset of female workers who were employed in the production of TCDD-contaminated phenoxy herbicides (Flesch-Janys et al. 1999; Kogevinas et al. 1993; Manz et al. 1991).

The results of the occupational cohort studies are supported by the most recent follow-up of the Seveso population (Consonni et al. 2008; Pesatori et al. 2009). On 10 July 1976, an explosion at a trichlorophenol manufacturing plant near Seveso, Italy, resulted in the highest TCDD levels known in human residential populations (Mocarelli et al. 1988). Up to 30 kg TCDD was deposited over the surrounding 18-km^2^ area (Di Domenico et al. 1980), which was divided into exposure zones (A, B, R, non-ABR) based on TCDD measurements in soil. After 20 years (1976–1996), cancer incidence was nonsignificantly increased in residents of the most exposed zone, zone A, for a wide range of cancer sites, including breast cancer (Pesatori et al. 2009). After 25 years (1976–2001), all cancer mortality was significantly increased in zone A when the analysis was limited to deaths occurring ≥ 20 years after the explosion (Consonni et al. 2008). Exposure estimates for these ecologic cohort studies were based on zone of residence and thus lacked individual-level exposure data.

The Seveso Women’s Health Study (SWHS), a historical cohort study of the female population residing around Seveso at the time of the explosion in 1976, represents the largest female population with known individual TCDD exposure (Eskenazi et al. 2000). Previously, using data from the SWHS, we examined the association between individual-level TCDD exposure, measured in archived serum collected soon after the explosion, and cancer risk 20 years later (Warner et al. 2002). We found a nonsignificant increased risk for all cancer incidence [hazard ratio (HR) = 1.7; 95% confidence interval (CI): 0.90, 3.4] and a significant increased risk for breast cancer incidence (HR = 2.1; 95% CI: 1.0, 4.8) associated with a 10-fold increase in individual serum TCDD levels. In 1996, however, the SWHS cohort was relatively young, averaging 40 years of age. Herein we report results for risk of cancer incidence with TCDD exposure from a subsequent follow-up of the cohort in 2008.

## Methods

*Study population.* The SWHS is a historical cohort study of the female population residing around Seveso at the time of the explosion in 1976. Details of the study design are presented elsewhere (Eskenazi et al. 2000). Briefly, eligible women were 0–40 years of age in 1976, had resided in one of the most highly contaminated zones, A or B, at the time of the explosion, and had adequate stored sera collected soon after the explosion. Enrollment began in March 1996 and was completed in July 1998 for study I. Of the 1,271 eligible women, 33 women were deceased or ill, 17 women were not reachable, and 240 refused to participate. A total of 981 (80%) participated in the first follow-up.

In 2008, we initiated a second follow-up study of the SWHS cohort (study II). Enrollment began in April 2008 and was completed in December 2009. Of the 981 eligible women, 16 (1.6%) were deceased and 36 (3.7%) could not be located or contacted. Of the remaining women who could be contacted, 833 (84.9%) agreed to participate.

*Procedure.* Details of the study procedure for the first follow-up study (study I) are presented elsewhere (Eskenazi et al. 2000). Briefly, participation included written informed consent, fasting blood draw, personal interview, and for a subset, a gynecologic examination and transvaginal ultrasound. Additional data were abstracted from medical records.

Participation in the second follow-up study (study II) included written informed consent, fasting blood draw, anthropometric and blood pressure measurements, personal interview, and for a subset, a bone density examination. Additional data were also abstracted from medical records.

For both study I and II, personal interviews were conducted by trained nurse-interviewers who were blinded to serum TCDD levels and zones of residence. During the interview, detailed information was collected on the reproductive and medical history of the women as well as demographic and lifestyle factors. Reproductive information collected included reproductive diseases, pregnancy history, history of hormone use, menopause status, medications use, and family history of cancer. Current information on other risk factors included use of cigarettes, alcohol or caffeine, calcium and other supplements, and social class factors (education, occupation, income). In addition, in study II, the European Prospective Investigation into Cancer and Nutrition (EPIC)–Italy food frequency questionnaire was administered (Pisani et al. 1997).

Medical history information obtained on the questionnaire included a diagnosis of cancer. During the interview, each woman was asked a series of cancer questions following the format, including “Has a doctor ever told you that you had breast cancer?” If she answered “yes” to any of the cancer questions, past medical records were obtained and were reviewed by a cancer pathologist who was blinded to the woman’s exposure. The death certificates were requested for all 16 deaths, and if cancer was indicated as a cause of death (*n* = 14), medical records were also requested. This study was approved by the institutional review boards of the participating institutions.

*Laboratory analyses.* TCDD was measured in archived sera by high-resolution gas chromatography/high-resolution mass spectrometry methods (Patterson et al. 1987). Values are reported on a lipid-weight basis in parts per trillion (ppt) (Akins et al. 1989). Details of serum sample selection are presented elsewhere (Eskenazi et al. 2000, 2004). Briefly, from the archived serum samples collected between 1976 and 1985 and stored at –20°C, we preferentially selected for analysis the first sample available that was collected between 1976 and 1981 and was of adequate volume (> 0.5 mL) to measure TCDD. We measured TCDD in sera collected in 1976 or 1977 for 894 women (91%), between 1978 and 1981 for 59 women (6%), and in 1996 or 1997 for 28 women (3%) with insufficient volume in earlier samples. For women with detectable post-1977 TCDD measurements > 10 ppt, the TCDD exposure level was back-extrapolated to 1976 using the first-order kinetic model (Pirkle et al. 1989) for women who were older than 16 years in 1976 (*n* = 40) or the Filser model (Kreuzer et al. 1997) otherwise (*n* = 30). For eight women whose post-1977 TCDD values were detectable but ≤ 10 ppt, the measured value was used. For nondetectable values (*n* = 96), a serum TCDD level of one-half the detection limit was assigned (Hornung and Reed 1990). For the study median serum sample weight of 0.65 g, the median limit of detection was 18.8 ppt, lipid-adjusted.

*Statistical analyses.* Serum TCDD was analyzed both as a continuous variable (log_10_TCDD) and a four-category variable. The cut point for the lowest group was set at ≤ 20 ppt, because 15–20 ppt was the average TCDD level in serum pools collected from unexposed Italian women in 1976 (Eskenazi et al. 2004). The three remaining categories were defined by calculating tertiles of exposure > 20 ppt, producing groups ≤ 20, 20.1–47.0, 47.1–135.0, and > 135 ppt.

We used Cox proportional hazards modeling for the main analyses. Age was the underlying time variable, with entry defined as the age of the subject on the explosion date, 10 July 1976, and exit defined as her age at cancer diagnosis or censoring (death, last follow-up). Women who refused or were not able to be located were censored at the age at last follow-up (1996–1998). We report the measure of effect as the HR and 95% CI.

We examined the effect of a broad range of potential confounders identified in the cancer and breast cancer literature (American Cancer Society 2010; Brody and Rudel 2003; Hulka and Moorman 2001; Key et al. 2001; Salehi et al. 2008; Travis and Key 2003). We considered education, marital status, gravidity, parity, age at first full-term pregnancy, lactation history, family history of breast cancer in a first-degree relative, age at menarche, body mass index (BMI), weight, height, oral contraceptive (OC) use, menarche status at explosion, time from explosion to first full-term pregnancy, age at explosion, menopause status, age at menopause, hormone replacement therapy use, history of thyroid disease, physical inactivity, smoking, and alcohol consumption. Covariate information for each woman was based on data collected at her last follow-up. We also considered age at menopause as a time-dependent variable. Covariates were included in Cox models if they changed the coefficient for log_10_TCDD by at least 10% or if they were independently associated with the cancer outcome at *p* < 0.10. We also considered possible interaction of menarche status at explosion.

In sensitivity analyses, we repeated the final models for cancer and breast cancer, stratifying on study follow-up period (study I: 1976–1996; study II: 1997–2009), on time between initial exposure and disease diagnosis or latency period (0–10 years; 11–20 years; 21–32 years), on menopause status at diagnosis, and on estrogen or progesterone receptor status. We also repeated the final models, excluding women whose individual TCDD level was measured in serum collected after 1977 and excluding women with individual TCDD levels derived by extrapolation. Finally, we repeated the final models assuming all nonparticipants (refusals, loss to follow-up) in study II were noncases.

Standard errors were estimated using the robust Huber–White sandwich estimator. The proportional hazards assumption was tested using scaled Schoenfeld residuals. We conducted tests for linear trend by including categorical TCDD as a continuous term in the models. All statistical analyses were performed using STATA 11.0 (version 11.0; StataCorp, College Station, TX, USA).

## Results

Table 1 presents the distribution of TCDD exposure by select characteristics of the SWHS cohort. On the date of the explosion, 232 women (24%) were < 10 years of age, and 284 (29%) were premenarcheal. At last follow-up, the average (± SD) age of the cohort was 50.8 (± 11.8) years, about half of the women (51%) were postmenopause, and 152 (16%) were nulliparous. The mean age at first pregnancy for the 829 parous women was 25.4 (± 4.6) years, and 711 (86%) had ever lactated. About 10% of women reported a family history of breast cancer in a first-degree relative (mother, sister, daughter). Most (63%) women had never regularly smoked or consumed alcohol. The average BMI at last follow-up was 26.4 (± 5.4) kg/m^2^, and 22% were obese. Serum TCDD levels were higher among women who were youngest or premenarche at explosion. TCDD levels were also higher among women who at last follow-up were nulliparous, current OC users, never married, more educated, or premenopause, but these are all also age-related characteristics.

**Table 1:** Distribution of 1976 serum TCDD levels (parts per trillion, lipid-adjusted) by select characteristics in the SWHS, Italy, 1976–2009.

				
				
				
				
				
				
				
				
				
				
				
				
				
**a**Numbers do not add to 100% of total because of missing data. **b**Parous women only. *p < 0.05 (ANOVA significant difference in log_10_TCDD by covariate).				

In total, 66 women (6.7%) in the SWHS cohort had been diagnosed with cancer. Of the 66 incident cases, 21 were diagnosed by the first follow-up (1996–1998), and 45 were diagnosed by the second follow-up (2008–2009). Of the 66 cases, 65 (98%) were confirmed by pathology and one (2%) by surgery report alone. The average age of cases (*n* = 66) at diagnosis was 48.8 (± 11.3) years and at explosion was 25.5 (± 10.9) years. The cases were diagnosed an average of 23.4 (± 7.2) years after the explosion, with the shortest interval being 7 years. The geometric mean (SD) serum TCDD level for the 66 cancer cases [95.3 (4.0) ppt, lipid-adjusted] is somewhat greater than the concentration for the noncases [*n* = 915; 67.9 (4.2)] [analysis of variance (ANOVA) for log_10_TCDD: *p* = 0.06].

The distribution of cancer sites among the 66 cases is presented in Table 2. Breast cancer (*n* = 33) was the most frequent site, representing half of the cases. Thyroid cancer (*n* = 7) was the second most frequent site. The remaining cases were represented by a wide variety of cancer sites.

**Table 2:** Distribution of cancer cases, SWHS, Italy, 1976–2009.

Cancer site (ICD-9 code)*a*	*n* (%)
All cancers	66 (100)
Digestive organs, peritoneum (150–159)	8 (12.1)
Esophagus (150)	1
Stomach (151)	2
Colon (153)	3
Rectum (154)	1
Other digestive (159)	1
Respiratory, intrathoracic organs (160–165)	2 (3.0)
Lung (162)	2
Bone, connective tissue, skin, breast (170–175)	36 (54.5)
Melanoma of skin (172)	3
Breast (174)	33
Genitourinary organs (179–189)	8 (12.1)
Cervix (180)	1
Placenta (181)	1
Uterus (182)	3
Ovary (183)	2
Kidney (189)	1
Other and unspecified sites (190–199)	9 (13.6)
Thyroid (193)	7
Ill-defined (199)	2
Lymphatic, hematopoietic tissue (200–208)	3 (4.5)
Lymphoma (202)	2
Myeloid leukemia (205)	1
**a***International Classification of Diseases, 9th Revision* (World Health Organization 1980).	

All breast cancers were confirmed by pathology. The average age of breast cancer cases at explosion was 26.4 (± 8.7) years, with a range from 10 to 39 years. The average age at diagnosis was 48.3 (± 8.5) years, with a range from 31 to 69 years. The average interval between explosion and diagnosis was 21.9 (± 7.3) years; the shortest interval was 8 years. Most of the 33 breast cancer cases (*n* = 18; 55%) were diagnosed premenopause. For the breast cancer cases for whom receptor status of tumors was available, 20 of 23 (87%) were estrogen receptor (ER)–positive, 19 of 22 (86.4%) were progesterone receptor (PR)–positive, and 6 of 12 (50%) were human epidermal growth factor receptor 2 (HER2)–positive. Of the 22 cases who had both ER and PR data, 82% were ER+PR+. There was no significant difference in receptor status of pre- and postmenopause cases.

Because of the small numbers of cases of specific cancer types, in multivariate analysis we were able to examine only the relation of serum TCDD levels to all cancers combined and to breast cancer. For thyroid cancer, in a univariate Cox model, the unadjusted HR associated with a 10-fold increase in TCDD (log_10_TCDD) was 2.1 (95% CI: 0.6, 6.7). Table 3 presents the unadjusted and adjusted results of Cox proportional hazards modeling for the association between lipid-adjusted serum TCDD level and all cancers combined and breast cancer risk.

**Table 3:** HRs from Cox proportional hazards model for association between lipid-adjusted serum TCDD levels and all cancer and breast cancer risk over 32 years of follow-up, SWHS, Italy, 1976–2009.

All cancers					Breast cancer				
Exposure	Cases/total	HR (95% CI)	Adjusted HR*a *(95% CI)	Cases/total	HR (95% CI)	Adjusted HR*b *(95% CI)
Log_10_TCDD*c* (ppt)		66/981		1.86 (1.34, 2.59)		1.80 (1.29, 2.52)		33/981		1.49 (0.93, 2.38)		1.44 (0.89, 2.33)
				*p *< 0.001		*p *= 0.001				*p = *0.10		*p *= 0.13
TCDD (ppt)												
≤ 20		6/154		1.00		1.00		4/154		1.00		1.00
20.1–47.0		14/276		1.16 (0.45, 3.02)		1.23 (0.48, 3.16)		7/276		0.91 (0.27, 3.08)		0.94 (0.28, 3.14)
47.1–135.0		25/278		2.53 (1.04, 6.18)		2.50 (1.02, 6.09)		13/278		2.07 (0.68, 6.30)		1.95 (0.64, 5.95)
> 135		21/273		2.92 (1.18, 7.24)		2.77 (1.11, 6.90)		9/273		2.04 (0.64, 6.48)		1.98 (0.62, 6.32)
				*p-*Trend = 0.001		*p-*Trend = 0.002				*p-*Trend = 0.07		*p-*Trend = 0.09
**a**Adjusted for marital status and age at explosion. **b**Adjusted for parity and family history of breast cancer in a first-degree relative. **c**HR for a 10-fold increase in serum TCDD concentration.												

For analysis of all cancers combined, in single-covariate Cox models cancer risk was positively associated with premenarche status at explosion, never married, current alcohol consumption, physical inactivity, lower gravidity, lower parity, and never lactating. As presented in Table 3, after adjusting for age at explosion and marital status, the adjusted HR associated with a 10-fold increase in TCDD (log_10_TCDD) for all cancers combined remained significantly increased to 1.80 (95% CI: 1.29, 2.52). When TCDD was considered as a categorical variable, there was still evidence of a significant dose–response trend (*p* = 0.002). Compared with the lowest exposure group (≤ 20 ppt), the adjusted HR (95% CI) for the three dose groups, 20.1–47 ppt, 47.1–135 ppt, and > 135 ppt, were 1.23 (0.48, 3.16), 2.50 (1.02, 6.09), and 2.77 (1.11, 6.90), respectively. We found no evidence of interaction by menarche status at explosion (*p* = 0.89). When stratified by zone, although the number of cases from zone A is small, we found no difference in risk of all cancers associated with serum TCDD levels by residence in zone A (*n* = 13; adjusted HR = 1.55; 95% CI: 0.86, 2.80) or zone B (*n* = 53; adjusted HR = 2.30; 95% CI: 1.32, 3.99). The *p*-value for an interaction of TCDD and zone was *p* = 0.27.

For the breast cancer analysis, in single-covariate Cox models, breast cancer risk was positively associated with younger age of menarche, lower gravidity, lower parity, never lactating, older age at first pregnancy, and family history of breast cancer. As presented in Table 3, after adjusting for parity and family history of breast cancer in a first-degree relative, the adjusted HR (95% CI) for breast cancer associated with a 10-fold increase in exposure (log_10_TCDD) was nonsignificantly increased to 1.44 (0.89, 2.33). When TCDD was categorized, there was some evidence of a dose response, but it was not statistically significant (*p* = 0.09). Compared with the lowest exposure group (≤ 20 ppt), the adjusted HR (95% CI) for the three higher dose groups were 0.94 (0.28, 3.14), 1.95 (0.64, 5.95), and 1.98 (0.62, 6.32), respectively.

If we consider the group “all cancers combined excluding breast cancer” (*n* = 33), the adjusted HR (95% CI) associated with a 10-fold increase in exposure (log_10_TCDD) was significantly increased to 2.08 (1.34, 3.23). When TCDD was categorized, there was evidence of a significant dose–response trend (*p* = 0.02). Compared with the lowest exposure group (≤ 20 ppt), the adjusted HRs (95% CI) for the three higher dose groups were 1.78 (0.38, 8.33), 3.29 (0.72, 14.92), and 3.96 (0.87, 18.24), respectively.

Table 4 presents the results of Cox proportional hazards models for all cancers and breast cancer, stratified by study follow-up period (study I: 1976–1996; study II: 1997–2009) and by latency period (0–10 years, 11–20 years, 21–32 years). Although the small numbers of cancer cases within strata limit power, the HRs presented for all cancer incidence do not differ by study follow-up period or latency period. For breast cancer, however, the significant increased risk reported for breast cancer incidence during study I is diminished in the last decade of follow-up and is no longer significant. Further, in sensitivity analyses, we found no difference in risk by menopause status at diagnosis or receptor status, but the numbers are small (data not shown).

**Table 4:** HRs from Cox proportional hazards model for association between lipid-adjusted serum (log_10_) TCDD levels and all cancer risk and breast cancer risk, stratified on follow-up period, SWHS, Italy, 1976–2009.

All cancers							Breast cancer						
Follow-up	Cases	p-y	Unadjusted HR*a *(95% CI)	*p*	Cases	p-y	Unadjusted HR*a *(95% CI)	*p*
1976–2009		66		29,722		1.86 (1.34, 2.59)		< 0.001		33		29,838		1.49 (0.93, 2.38)		0.10
Study I (1976 to 1996–1998)		21		20,118		1.71 (0.93, 3.14)		0.08		15		20,168		2.13 (1.11, 4.09)		0.02
Study II (1996–1998 to 2008–2009)		45		9,604		1.83 (1.25, 2.67)		0.002		18		9,670		1.12 (0.61, 2.06)		0.71
0–10 years (1976–1986)		6		9,802		2.39 (1.13, 5.06)		0.02		3		9,808		2.91 (0.90, 9.44)		0.08
11–20 years (1987–1996)		13		9,699		1.61 (0.72, 3.59)		0.25		10		9,737		2.23 (1.09, 4.56)		0.03
21–32 years (1997–2009)		47		10,221		1.77 (1.21, 2.58)		0.003		20		10,293		1.06 (0.58, 1.93)		0.86
p-y, person-years. **a**HR for a 10-fold increase in serum TCDD concentration.																

We repeated the final models, first limiting the analysis to the 894 women with TCDD measured in samples collected in 1976 or 1977, and then excluding 78 women with extrapolated TCDD measures, and the results were not different (data not shown). Finally, we assumed all nonparticipants (refusals, loss to follow-up) were noncases, and the results did not change (data not shown).

## Discussion

We observed a statistically significant, dose-related increased risk in overall cancer incidence associated with individual serum TCDD level in the SWHS. Specifically, we reported a significant increased HR of 1.8 associated with a 10-fold increase in serum TCDD. This result is similar to our previous observation, although the association was not statistically significant in the earlier follow-up in 1996–1998 (Warner et al. 2002).

The validity of the findings is strengthened by the fact that the results did not change and remained significant after adjusting for potential confounding factors. Participation in study II was high (> 85%) 12 years after study I and > 30 years after the explosion. In addition, loss to follow-up was low (3.7%). Participants and nonparticipants (refusals, loss to follow-up) did not differ in terms of age at explosion or serum TCDD level.

The results of this study are consistent with those from earlier studies suggesting an association but lacking individual exposure data. The two occupational cohort studies of workers employed in the production of chlorophenoxy herbicides both reported increased risks for mortality and incidence from all cancers in the subset of female workers who were employed in the production of TCDD-contaminated phenoxy herbicides (Flesch-Janys et al. 1999; Kogevinas et al. 1993).

The results are also consistent with those reported for zone A, but not zone B, in the most recent cancer incidence study of the larger Seveso population (Pesatori et al. 2009). After 20 years (1976–1996), nonsignificant increased risks for cancer incidence were reported in zone A for a wide range of cancers. In zone A, after a 15-year latency, all cancer incidence for males and females together was nonsignificantly increased (*n* = 19; rate ratio (RR) = 1.27; 95% CI: 0.81, 2.00), but sex-specific risks were not presented. Our results, however, are not consistent with those reported for zone B, which, in contrast to zone A, after a 15-year latency had no increase in all cancer incidence for males and females together (*n* = 92; RR = 1.02; 95% CI: 0.83, 1.26). The lack of consistency for zone B is likely attributable, in part, to exposure misclassification. Individual serum TCDD measurements from the SWHS suggest a wide range of individual TCDD exposures within zones (Eskenazi et al. 2004). Exposure misclassification based on zone of residence would be expected to be nondifferential, potentially resulting in an underestimate of effect. Although the number of cases from zone A is small in our study, we found no difference between zone A and zone B in risk of cancer associated with serum TCDD levels.

The results are also somewhat consistent with the most recent cancer mortality study of the larger Seveso population (Consonni et al. 2008). After 25 years of follow-up (1976–2001), the number of cancer deaths in zone A was small (*n* = 42), but with a 20-year latency, mortality from cancer among zone A women was nonsignificantly increased (*n* = 5; RR = 1.17; 95% CI: 0.49, 2.83). Similar data were not presented for zone B women. There is likely little overlap in cases between the mortality study and SWHS. The SWHS cohort included women who were 0–40 years of age in 1976, whereas the larger Seveso cohort included women who were 20–74 years of age. In addition, we included incident cases diagnosed between 1976 and 2009; 14 of the SWHS women were deceased, but only one case died before 2001, the end of follow-up for the mortality study (Consonni et al. 2008).

An advantage of SWHS is that we were able to examine the relationship between serum TCDD concentration and cancer incidence, not mortality, thus eliminating potential biases associated with variations in disease survival. In addition, we were able to collect information on confounding factors during the interview that were not available in the mortality study. Finally, we were able to measure individual serum TCDD concentrations near the time of exposure, thus minimizing exposure misclassification.

A limitation of the SWHS study is the small number of cancer cases (*n* = 66). However, this number is greater than the number in any other single study of cancer in TCDD-exposed women (Flesch-Janys et al. 1999; Kogevinas et al. 1993; Pesatori et al. 2009). These other studies, with numbers of cases ranging from 9 to 57, reported increased cancer risks similar to the HRs reported here. These studies classified exposure based on job history, company production records, and, for a subset of workers, TCDD in serum or adipose measured many years after last exposure.

Although we observed a wide range of cancers in this study, only breast cancer had enough cases for an examination of the association with TCDD. We found a nonsignificant increase in the HR (HR = 1.44; 95% CI: 0.89, 2.33) associated with a 10-fold increase in serum TCDD over the 32-year follow-up period. Our earlier observation of a significantly increased risk of breast cancer with 20 years of follow-up (Warner et al. 2002) was not sustained with the additional 12 years of data. In categorical analysis, although not significant, the highest risks were observed for the highest dose groups.

The results for breast cancer in this study are somewhat consistent with those from the 20-year incidence study of the larger Seveso population, which reported a nonsignificant increased risk for breast cancer in zone A (*n* = 8; RR = 1.43; 95% CI: 0.71, 2.87), but not zone B (*n* = 30; RR = 0.85; 95% CI: 0.59, 1.22) (Pesatori et al. 2009). Pesatori et al. (2009) found no difference in risk by age at diagnosis of breast cancer before or after 50 years of age, a proxy for menopause status (< 50: *n* = 3; RR = 1.50; 95% CI: 0.48, 4.67 vs. ≥ 50: *n* = 5; RR = 1.39; 95% CI: 0.58, 3.36). Consistent with that report, we found no difference in risk of breast cancer with TCDD exposure by menopause status at diagnosis. The two studies, however, are not directly comparable. The SWHS cohort is younger; at explosion, the 33 breast cancer cases in SWHS averaged 26.4 years of age, whereas the 8 breast cancer cases in zone A in the study by Pesatori et al. (2009) were 20–49 years of age.

When we compared the observed breast cancer incidence in the SWHS cohort with that expected for the area using 1985–2009 age-specific breast cancer incidence rates from the Lombardy Cancer Registry in Italy (Italian Association of Cancer Registries 2010), the expected number of breast cancer cases would be 27; we found 33 for an overall standardized incidence ratio for the 981 women of 1.22. Further, the observed excess of cases was highest in the 45- to 49-year age group (10 observed vs. 5 expected).

Of the breast cancer cases for whom receptor status data were available, the distribution of ER+ and PR+ but not HER2+ was similar to that reported by the Tuscany Cancer Registry among all the invasive breast cancer cases diagnosed during the period 2004–2005 (Caldarella et al. 2011). Given the limited number of cases (*n* = 12) with HER2+ data available for SWHS, however, it is difficult to determine whether there is any difference in receptor status.

It is possible the increased risk for breast cancer observed in earlier decades of follow-up reflects a mechanism of action of TCDD as a cancer promoter. It is also worth noting that the cohort has not yet been followed to the time of greatest onset of breast cancer—postmenopause. With additional follow-up, we will be able to better discern whether the window of increased risk for breast cancer observed in the earlier follow-up period is in fact past.

Additional follow-up is also needed for the observed cancers other than breast. There is evidence in animals of an effect of TCDD on thyroid function, including an increase in thyroid follicular cell hyperplasia (Nishimura et al. 2002, 2003; Yoshizawa et al. 2010). The number of thyroid cancer cases is small, but it is noteworthy that the observed increase in HR is consistent with the animal studies. We also observed two cases of lung cancer (one in a nonsmoker) and three cases of lymphatic and hematopoietic cancers, both types of cancer previously reported to be associated with TCDD exposure (Consonni et al. 2008; IARC 1997; Pesatori et al. 2009), but we were unable to examine the relation of TCDD because of the small number of cases.

In summary, we have shown that individual serum TCDD measurements are significantly positively related to overall cancer incidence among women in the SWHS cohort. The results of this study are consistent with TCDD as a potent multisite carcinogen in animals and with increased cancer mortality risks reported in the male occupational cohort studies and used by IARC in its classification of TCDD (IARC 1997; Steenland et al. 2004). Thus, this study extends the results of the recent IARC reassessment to include women. SWHS women were 0–40 years of age at exposure, and almost one-third of the women were premenarche at exposure. With continued follow-up of SWHS, we will begin to be able to examine the carcinogenic effects of TCDD during potentially susceptible windows of exposure such as premenarche or time between menarche and age at first full pregnancy.
